# Comparative Study on Micro-Grinding Performance of 2.5D C_f_/SiCs, 2.5D SiC_f_/SiCs, and SiC Ceramics

**DOI:** 10.3390/ma16196369

**Published:** 2023-09-23

**Authors:** Quan Wen, Yuanfeng Li, Yadong Gong

**Affiliations:** School of Mechanical Engineering & Automation, Northeastern University, Shenyang 110819, China

**Keywords:** 2.5D C_f_/SiC composites, 2.5D SiC_f_/SiC composites, SiC ceramics, micro-grinding, surface micro-topography, surface roughness, grinding force

## Abstract

To investigate the micro-grinding process and performance of 2.5D C_f_/SiC composites and 2.5D SiC_f_/SiC composites in depth, single-factor micro-grinding experiments were conducted by using SiC ceramics as a comparison. Differences in the material removal process, surface microstructure, surface roughness, and grinding force of the three materials under the same grinding parameters were comparatively analyzed. The results indicate that crack propagation is severe during the micro-grinding process of SiC ceramics. The ground surface is uneven, accompanied by pit defects and large surface roughness *Ra*. However, the presence of reinforcing fibers and interfaces in the two types of composites can inhibit crack propagation or change their extension directions. Therefore, their surfaces are smooth and flat after grinding, with small defects and low surface roughness *Ra*. In addition, the grinding processes of the two composites are both related to fiber orientation. There are differences in crack propagation paths and fiber fracture positions in the weft fiber layer and the radial fiber layer, which result in different forms of grinding defects. During micro-grinding, the real-time force signals of 2.5D C_f_/SiC composites and 2.5D SiC_f_/SiC composites are relatively stable, while the signals of SiC ceramics have a large number of spikes. The average micro-grinding force of the three materials is: SiC ceramics > 2.5D SiC_f_/SiC composites > 2.5D C_f_/SiC composites.

## 1. Introduction

In harsh environments such as extreme high temperatures, radiation, and chemical reactions, metals and alloys are no longer able to meet requirements. Ceramic materials with the advantages of high temperature resistance and good stability in extreme environments are eye-catching [1]. However, the brittleness and high hardness of ceramic materials limit their widespread application [2]. In recent years, some researchers have woven fibers into the ceramic matrix, which not only maintains the advantages of ceramics, but also increases their toughness. In this way, fiber-reinforced ceramic matrix composites have been widely used in extreme harsh environments such as aerospace, thermal structure, atomic energy, and other fields [3,4,5].

Composite materials often require extensive mechanical processing after preparation and molding. The harsh application environments impose strict requirements on the surface quality of fiber-reinforced ceramic matrix composites. However, their characteristics of high hardness, high brittleness, multiphase structure, and anisotropy make them prone to various defects during machining, resulting in poor surface quality [6]. As new types of materials, their machining performance has received extensive attention. At present, compared with non-traditional machining methods, grinding with a diamond wheel is a precision machining method widely used for ceramic matrix composites because of its high machining efficiency and low production cost [7].

In recent years, many studies have focused on the grinding process, material removal mechanism, surface integrity, and grinding force of ceramic matrix composites. Most of them revolve around C_f_/SiC composites. Zhang et al. [8] investigated the grinding process of unidirectional C_f_/SiC composites. Analysis of the experimental results shows that brittle fracture is the dominant removal mechanism for grinding of the C/SiC composites, and the destroyed form of the composites is mainly the syntheses of the matrix cracking, fiber fracture, and interfacial debonding. A special rigid foundation beam model was established by Qu et al. based on the properties of the unidirectional C_f_/SiC composites [9]. The evolutions of the grinding forces and debonding depth can be derived based on the mechanical model. Du et al. [10] studied the effect of machining parameters on cutting force, force ratio, and 3D surface roughness on the grinding of 2D C/SiC composites. Two machining directions on one surface were taken into account to study the effect of fiber orientation on the grinding process and machinability. Guo et al. [11] proposed a single abrasive particle grinding finite element simulation model, which considers material anisotropy and an interface constitutive model. The effects of fiber orientation, interface strength, and grinding parameters on the grinding performance and surface quality of C_f_/SiC composites were analyzed through the model. Chen et al. [12] explored the material removal mechanism of C_f_/SiC composites in grinding through the different fracture mechanisms of carbon fibers from the perspective of microstructure. It was reported that the difference in machined surface morphology was directly caused by the difference in material removal mechanism, which can be effectively characterized by the machined surface roughness. Azarhoushang [13] designed segmented diamond wheels to remove carbon-fiber-reinforced ceramic composites in high-speed deep grinding, which showed a better wheel wear resistance. Liu proposed a method of laser-grinding chain processing C/SiC composite groove. The method combined the high efficiency characteristic of laser ablating and the high precision characteristic of grinding. The appropriate laser processing parameters and grinding parameters were optimized [14]. Gong et al. [15] compared the machinability of 2.5D C_f_/SiC composites and SiC materials by utilizing a diamond grinding wheel with 200 mm diameter. They pointed out that the extension of cracks is the main removal method during the grinding process of SiC materials, while matrix craze, fiber fracture, and interfacial debonding are the main removal methods of C_f_/SiC composites.

In addition, a small amount of research has focused on the grinding of SiC_f_/SiC composites. Ran et al.’s research suggests that the grinding force of the SiC_f_/SiC composites exhibits a significant correlation with fiber orientation [16]. This is attributed to the fact that the fiber orientation influences the crack propagation path; meanwhile, the complex and diverse crack propagation paths make the degree of fluctuation of grinding force and the energy required for material removal vary along different fiber orientations. Luna et al. [17] carried out scratch tests in a circular trajectory with a single abrasive grain with different geometries and sizes, and arrays of overlapped grains. They reported that the crack onset location is governed by the grain shape, but its direction of propagation depends on the fiber orientation. Zhang et al. [18] developed a side grinding model considering the anisotropy of orthogonal laminated SiC_f_/SiC composites and the fracture removal mechanism of the brittle material. The grinding process was divided into the ductile, ductile-to-brittle transition, and brittle stages for analysis by the critical cutting depth. Liu et al. [19] investigated the scribing force and material removal mechanism of 2.5D woven SiC_f_/SiC with flat and sharp diamond grits. They discovered that the scribing surface showed many kinds of removal mechanisms.

Except for mechanical processing, scratching experiments are also an effective means to study the mechanisms of material removal. Zhang et al. [20] conducted an orthogonal test of single-grain scratches under different lubrication modes. They pointed out that the dominant material removal mode of the SiC_f_/SiC composites was a brittle fracture, and the damage characteristics mainly included natural fiber fracture, matrix breakage, fiber pullout, and fiber exposure. Lin et al. [21] analyzed the mechanism of material removal in ultrasonic-vibration-assisted scratching of 2D SiC_f_/SiC composites. In the ultrasonic-assisted scratching process, the surface roughness is improved because the fiber undergoes a brittle fracture, the matrix is torn, and the surface residues are discharged in time. Wang et al. studied the material removal of unidirectional C_f_/SiC composites. Their results showed that the parallel scratching process could produce a better strength resistance with mild rupture features of fiber extrusion fracture and shear removal, while the perpendicular scratching process generally results in crack propagation along the fiber direction with interface failure and bending fracture failure [22].

According to the currently published literature, most of the relevant studies focus on the conventional-scale grinding of unidirectional or two-dimensional ceramic matrix composites. There are few studies on micro-scale grinding of 2.5-dimensional carbon-fiber-reinforced ceramic matrix composites (2.5D C_f_/SiCs) and 2.5-dimensional silicon carbide fiber-reinforced ceramic matrix composites (2.5D SiC_f_/SiCs). At present, the cost of manufacturing large structural 2.5D C_f_/SiCs and 2.5D SiC_f_/SiCs remains high since their preparation processes are relatively complex, but they have good application prospects in the field of manufacturing micro parts. Additionally, due to factors such as size effects, micro-grinding differs from conventional-scale grinding. It is not simply a proportional reduction of the conventional-scale grinding process [23,24].

To meet the requirements of material surface quality for micro critical parts, the micro-grinding performance of 2.5D C_f_/SiCs and 2.5D SiC_f_/SiCs were explored in this article. Single-factor experiments were conducted by using micro-grinding tools with diameters of 0.9 mm. To better understand the effects of reinforcing fibers and interfaces on the machinability of ceramic matrix composites, comparative experiments were conducted on SiC ceramics under the same grinding parameters. Differences in micro-grinding process and grinding performance evaluation parameters (including surface microstructure, surface roughness, and grinding force) of the three materials were compared. Comparative analyses were conducted on the material removal mechanisms of crack generation and propagation during micro-grinding of these three materials, as well as the defect generation mechanisms. This research could provide some theoretical guidance for understanding the performance of these materials and achieving high-quality ceramic matrix composite micro-grinding.

## 2. Experimental Methods and Equipment

### 2.1. Experimental Materials, Micro-Grinding and Measuring Equipment

**Figure 1:**
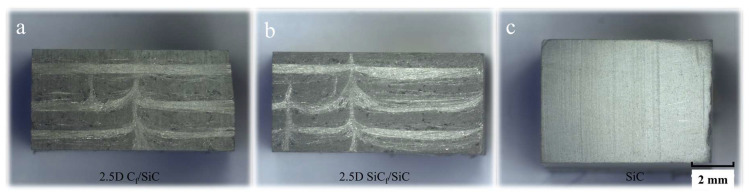
Morphologies of the three material samples.

**Figure 2:**
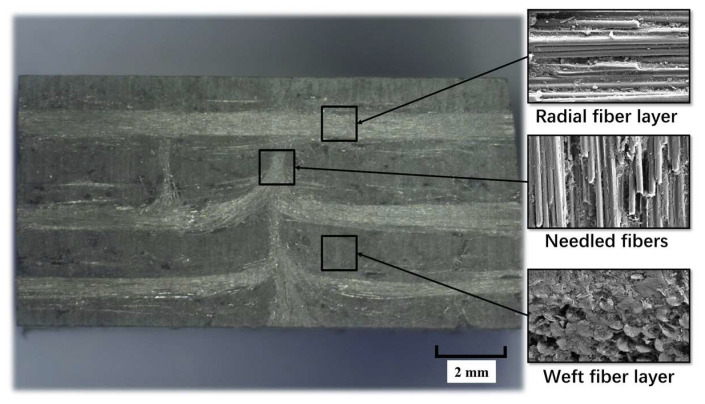
Microscopic morphology of the cross-section of 2.5D C_f_/SiCs.

The experimental materials are 2.5D C_f_/SiCs, 2.5D SiC_f_/SiCs, and SiC ceramics. The morphologies of the three materials are shown in Figure 1. It can be seen that the microstructures of the first two composite materials are similar. Their differences lie in the types of reinforcing fibers. The reinforcing fiber in Figure 1a is carbon fiber, while the reinforcing fiber in Figure 1b is silicon carbide fiber. The microscopic morphology of the cross-section of 2.5D C_f_/SiCs is shown in Figure 2. Under scanning electron microscopy, the characteristics of radial fibers, weft fibers, and needle-punched structures can be observed. They are distributed in the SiC ceramic matrix.

In the process of preparing the two composite materials, the preform needs to be prepared first. It was fabricated by alternatively stacked weftless plies and short-cut-fiber webs using a needle-punching technique. Two successive plies were oriented at an angle of 90°. The processing temperature was increased to 1200 °C and kept there for 2 h in a vacuum furnace. Secondly, in order to produce a pyrocarbon interphase and increase the thermal conductivity, a small amount of pyrocarbon was added to the treated preform by pyrolyzing natural gas for 10 h at 1000 °C in an isothermal chemical vapor infiltration apparatus. Finally, the treated preforms, clamped between two graphite electrodes for direct heating by passing an electric current in a 50 kW coldwall and normal-pressure furnace, were rapidly densified by forming a SiC matrix with the heaterless chemical vapor infiltration technique. Detailed preparation methods can be found in references [25,26,27].

**Figure 3:**
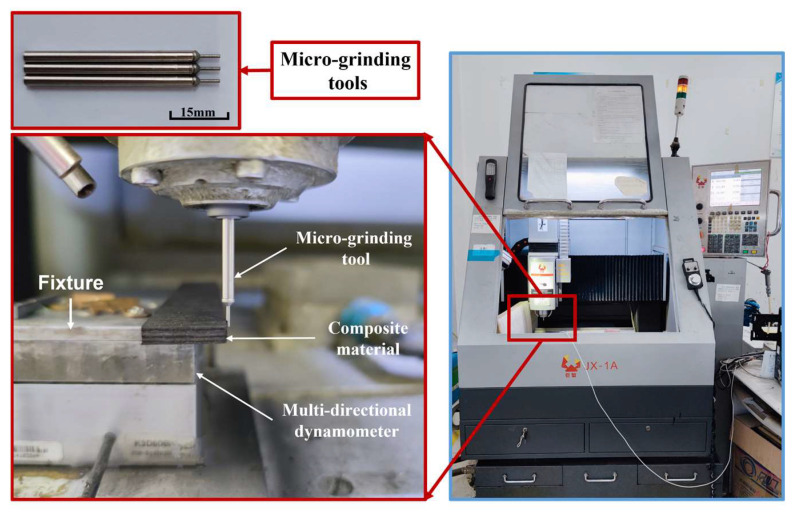
Micro-grinding experimental platform.

Experiments were carried out on the micro-grinding experimental platform, as shown in Figure 3. The platform includes a JX-1A precision grinding machine whose positioning resolution is 0.1 μm. The spindle speed of the machine could achieve 60,000 r/min. Micro-grinding tools were electroplated with 500# diamond abrasive particles with diameters of 0.9 mm. During the experiments, grinding forces were measured using a Kistler 9257B three-dimensional dynamometer (Kistler, Winterthur, Switzerland). The electrical force signals of the dynamometer were converted into numerical signals by a charge amplifier and a data collector, which were then processed by a computer.

After micro-grinding experiments, an Olympus OLS41003D laser confocal microscope (Olympus, Tokyo, Japan) was used to measure the surface roughness of the ground materials. Their surface morphologies were observed by a Kenyence VHX-1000E ultra-depth-of-field microscope (Kenyence, Osaka, Japan) and a Zeiss Ultra Plus field emission scanning electron microscope (Zeiss, Oberkochen, Germany).

### 2.2. Experimental Factors and Levels

Single-factor micro-grinding experiments were conducted on 2.5D C_f_/SiCs, 2.5D SiC_f_/SiCs, and SiC ceramics. Differences in the surface microstructure, surface roughness, and grinding force of the three materials under the same grinding parameters (grinding speed *v*_s_, grinding depth *a*_p_, and feed speed *v*_w_) were compared. The influence of various grinding parameters on the grinding performance was also explored. The experimental plan is shown in Table 1. The cutting speeds of 0.471, 0.942, 1.414, 1.885, and 2.356 m/s correspond to the spindle speeds of 10,000, 20,000, 30,000, 40,000, and 50,000 r/min, respectively.

**Figure 4:**
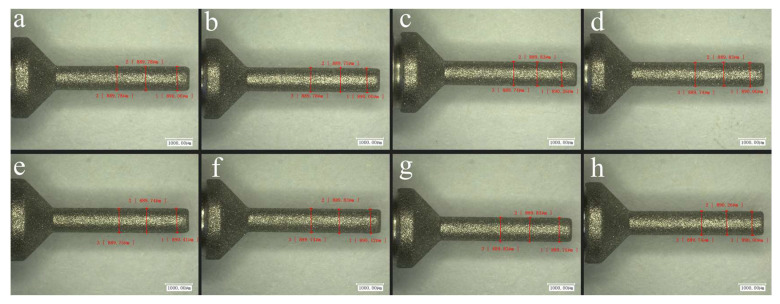
Measurement of the diameters of unused micro-grinding tools (**a**–**h**).

**Figure 5:**
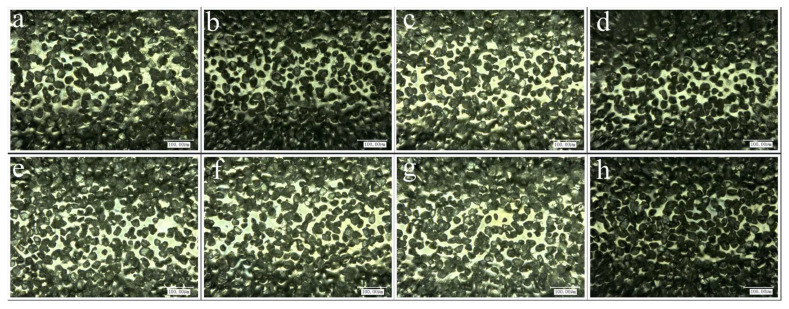
Surface morphologies of unused micro-grinding tools (**a**–**h**).

In the experiment, the experimental error was reduced through the following aspects. Firstly, in micro-grinding, the grinding depth *a*_p_ is as small as a few micrometers to over ten micrometers. In order to accurately ensure the actual grinding depth in the experiments, a 3 mm diameter grinding tool was used to pre-grind the material samples. After numerous instances of pre-grinding and testing to ensure sufficient flatness of the machined surface, formal micro-grinding experiments were carried out according to the experimental schemes in Table 1. Secondly, five parallel experiments were conducted on each set of grinding parameters to reduce random errors. The average of the five measured values of the grinding forces and surface roughnesses were taken as the test results under this set of parameters. Finally, the diameter of the micro-grinding tool was only 0.9 mm. Considering that the wear of the tool may affect the experimental results, the tool was replaced after the experiment on each set of parameters was conducted. Eight micro-grinding tools were randomly selected from the unused tools and were observed under the microscope. Figure 4 and Figure 5 show the diameters and surface morphologies of the tools, respectively. It can be seen that the consistency of the characteristics of these grinding tools is good.

## 3. Results and Discussion

### 3.1. Comparative Analysis of Grinding Process and Surface Morphology of the Three Materials

**Figure 6:**
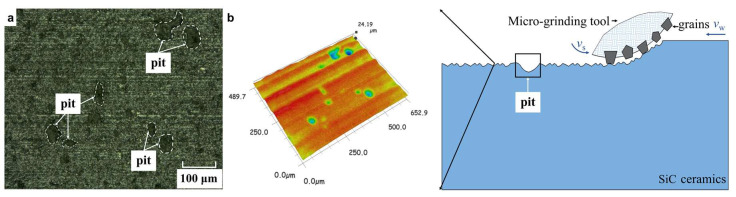
Surface morphology and material removal process of SiC ceramics (**a**,**b**).

**Figure 7:**
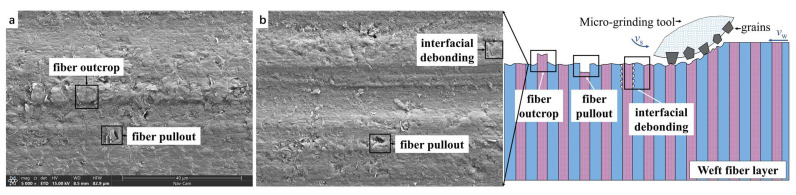
Surface morphology and material removal process of weft fiber layer of the two composites (**a**,**b**).

**Figure 8:**
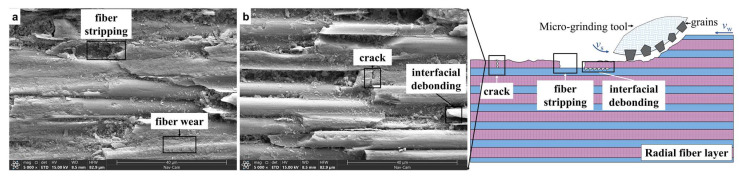
Surface morphology and material removal process of radial fiber layer of the two composites (**a**,**b**).

Comparative observations were conducted on the surface microstructures of the three materials after micro-grinding under the same parameters (*v*_s_ = 1.414 m/s, *a*_p_ = 15 μm, *v*_w_ = 120 μm/s). Since the surface morphologies of SiC ceramics and the two kinds of fiber-reinforced ceramic matrix composites are quite different, they were observed at different magnifications to clearly show their microscopic characteristics. For SiC ceramics, their microstructure was obtained using a Kenyence VHX-1000E ultra-depth-of-field microscope at a magnification of 500 times as shown in Figure 6. For the two composites, their microstructures were obtained using a Zeiss Ultra Plus field emission scanning electron microscope at the magnification of 5000 times as shown in Figure 7 and Figure 8.

From Figure 6a,b, it can be seen that the surface of SiC ceramics after micro-grinding is uneven with some obvious pit defects. SiC ceramics are typical brittle materials. The removal process of the brittle materials involves the abrasive particles acting on the material surface, causing microcracks to occur. The microcracks continue to expand and intersect, resulting in the material being removed [28]. According to the grinding mechanism of the brittle materials and the surface microstructure of SiC ceramics after micro-grinding in Figure 6, the removal process of SiC ceramics undergoes the following stages.

Firstly, when the abrasive particle on the micro-grinding tool just comes into contact with the SiC ceramics, a minimal elastic deformation occurs in the material, followed by plastic deformation. Then, as the abrasive particle further penetrates, the cutting thickness of the abrasive particle is greater than the critical cutting thickness of the ceramic, and microcracks occur in the material. Under the load of the abrasive particles, microcracks further develop into transverse and longitudinal cracks. These cracks propagate irregularly inside the material. Finally, when the transverse and longitudinal cracks intersect at a certain point, or when the transverse crack extends to the material surface, the SiC ceramics are removed. SiC ceramics are homogeneous materials that lack the ability to suppress crack propagation internally. Therefore, some cracks will extend further or intersect deeper below the surface, resulting in severe concave defects after the materials are removed.

For the two types of composite materials, the micro-grinding tool acts on both the weft fiber layers and radial fiber layers simultaneously. The surface microstructures after micro-grinding of these two types of fiber layers are quite different, so they will be discussed separately below.

Figure 7a,b show the surface microstructures of the weft fiber layers of 2.5D C_f_/SiCs and 2.5D SiC_f_/SiCs after micro-grinding, respectively. It can be seen that the surface morphologies of the two composites are similar. This is because their compositional structures are similar. Their matrices are both SiC ceramics, with only the types of reinforcing fibers differing. After micro-grinding, most of the SiC matrix and fiber fracture surfaces of the two composites are relatively flat. However, there are still some minor defects such as fiber pull-out, fiber outcrop, and interfacial debonding.

As multiphase materials, the removal process of the two composites during micro-grinding is different from that of homogeneous SiC ceramics mentioned above. When the abrasive particle comes into contact with the SiC matrix, the process of deformation and microcracking of it is similar to the process of SiC ceramics described earlier. That is, microcracks develop into transverse and longitudinal cracks under the load of the abrasive particle. The difference is that when transverse and longitudinal cracks propagate to the interfacial layer between the matrix and fibers, they usually interrupt or deflect due to the relatively poor mechanical properties of the interfacial layer. The matrix is removed after a short distance of crack propagation. As the abrasive particles further feed, some cracks will expand to the fiber-reinforced phase. Because the diameters of the C fiber and SiC fiber are only about 7 μm, the crack quickly extends to the interfacial layer again. The crack breaks or deflects along the interfacial layer again, causing the fibers to be removed. The presence of reinforcing fibers and interfaces has an inhibitory effect on the crack propagation. Therefore, the surfaces of both 2.5D C_f_/SiCs and 2.5D SiC_f_/SiCs after micro-grinding are much smoother compared to the SiC ceramics.

In addition, the distribution of fiber strength and the direction of crack propagation are random to a certain extent. As illustrated in Figure 7, some transverse cracks propagate into the interior of the material, causing the fracture position of the fibers to be lower than the grinding plane. After such fibers are pulled out, small holes are left, leaving fiber pulled out defects. Some transverse cracks propagate towards the material surface, causing the fracture position of the fibers to be higher than the grinding plane. After these fibers are removed, fiber outcrop defects are left. A portion of longitudinal cracks propagate along the interfacial layer and terminate, resulting in interfacial debonding defects.

The surface microstructures of the radial fiber layers of 2.5D C_f_/SiCs and 2.5D SiC_f_/SiCs after micro-grinding are shown in Figure 8a,b. Most surfaces are relatively flat, but there are still some minor defects such as fiber stripping, interfacial debonding, and fiber crack.

For the micro-grinding of the radial fiber layer, the removal process of the SiC matrix is similar to that of the weft fiber layer mentioned above. Similarly, due to the inhibitory effect of reinforcing fibers and interfaces on crack propagation, the surface of the radial fiber layer after micro-grinding is also smoother than that of the homogenous SiC ceramics. But, the fracture mode of fibers and the grinding defects are different from those of the weft fiber layer.

When grinding C or SiC fibers in the radial fiber layer, cracks may occur inside the fibers. Among these cracks, some of them propagate to the interfacial layer and interrupt, leaving fiber crack defects. Some of them propagate to the interfacial layer and then deflect. They continue to extend along the interfacial layer for a certain distance before stopping, resulting in interfacial debonding defects. Some of them extend along the interfacial layer for a certain distance and deflect again in areas with weaker fiber strength, causing the fibers to be peeled off as a whole. After these fibers are peeled off, shallow pits are left on the ground surface, which are fiber-stripping defects. In the single-particle scratch experiment on SiC_f_/SiC composite materials, when the angle between the scratching direction and the fiber direction was 0°, similar defects such as fiber pullout and interface debonding were also observed [20].

### 3.2. Comparative Analysis of Surface Roughness of the Three Materials

**Figure 9:**
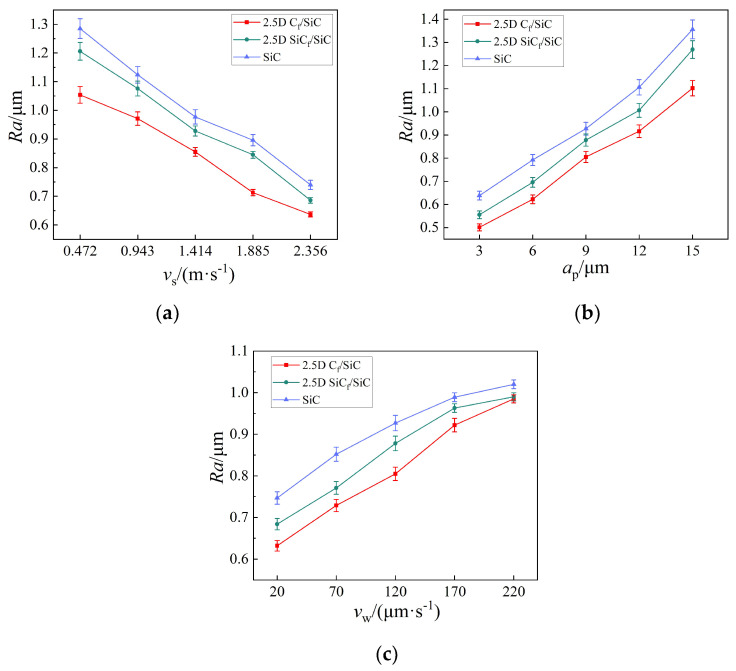
Influence of grinding parameters (**a**) grinding speed *v*_s_; (**b**) grinding depth *a*_p_; and (**c**) feed speed *v*_w_ on surface roughness *Ra* of the three materials.

Surface roughness of the material is an important indicator for quantitatively evaluating its grinding quality. The surface roughness *Ra* of the three materials after micro-grinding was measured by an Olympus OLS41003D laser confocal microscope. As introduced in Section 2.2, five parallel experiments were conducted on each set of grinding parameters to reduce the random errors. Through measurements and statistics, it was determined that the deviations between the measured surface roughness values of each experiment and their average values were within ±3%. The average values are listed in Table 2. In order to express the results clearly and intuitively, the data in Table 2 are plotted as line charts in Figure 9.

For the three materials, the influence of grinding parameter changes on surface roughness was basically consistent. As shown in Figure 9a, when the grinding depth *a*_p_ and the feed speed *v*_w_ were 9 μm and 120 μm/s, respectively, and the grinding speed vs. increased from 0.471 m/s to 2.356 m/s, the surface roughness *Ra* of 2.5D C_f_/SiCs, 2.5D SiC_f_/SiCs, and SiC ceramics decreased by 41.5%, 45.0%, and 44.1%, respectively. This is because an increase in speed increases the number of abrasive particles involved in grinding per unit time. The repeated grinding of more abrasive particles on the material surface results in a smoother surface.

In addition, as shown in Figure 9b, as vs. and *v*_w_ were 1.414 m/s and 120 μm/s, respectively, and *a*_p_ increased from 3 μm to 15 μm, the surface roughness *Ra* of the three materials after grinding increased by 119.9%, 128.6%, and 112.5%, respectively. This is because the cutting depth of a single abrasive increases with an increase in *a*_p_. The interaction surface between the abrasive and the material also increases. So, more microcracks will be generated on the interaction surface. With the propagation and intersection of the microcracks, the materials are removed and more defects are left on the ground surface, resulting in an increase in the surface roughness *Ra*.

Additionally, as shown in Figure 9c, as vs. and *a*_p_ were 1.414 m/s and 9 μm, respectively, and *v*_w_ increased from 20 μm/s to 220 μm/s, the surface roughness *Ra* of the three materials after grinding increased by 55.8%, 44.7%, and 36.5%, respectively. This is because as *v*_w_ increases, the impact and force generated by the interaction between a single abrasive particle and the material are greater. Under the impact of the abrasive particle, the material generates more microcracks, which will leave more defects on the machined surface and increase its roughness *Ra*.

As shown in Figure 9a–c, under the same grinding parameters, *Ra* of 2.5D C_f_/SiCs is lower than that of 2.5D SiC_f_/SiCs, while both of them are lower than that of SiC ceramics. This can be explained by the analysis of the material structure and crack propagation mode of the three materials. During the micro-grinding process, the presence of reinforcing fibers in the composites can suppress the random propagation of cracks. Transverse cracks and longitudinal cracks either stop or deflect at the interface layer to remove the material, or they intersect at a close point to remove the material, or transverse cracks reach the material surface to remove the material. Regardless of which of the three situations mentioned above applies, the paths of the crack propagation are all relatively short. Therefore, the surface of the ground material is flat and the *Ra* value is low. However, during the removal process of SiC ceramics, because the random propagation of cracks cannot be suppressed, some transverse and longitudinal cracks intersect at a farther distance or a deeper depth within the material. After such materials are removed, the surface of SiC ceramics will be uneven and even leave serious pit defects. As a result, compared to the two types of fiber-reinforced composites, the surface roughness value *Ra* of the SiC ceramics after micro-grinding is higher.

### 3.3. Comparative Analysis of Grinding Forces of the Three Materials

**Figure 10:**
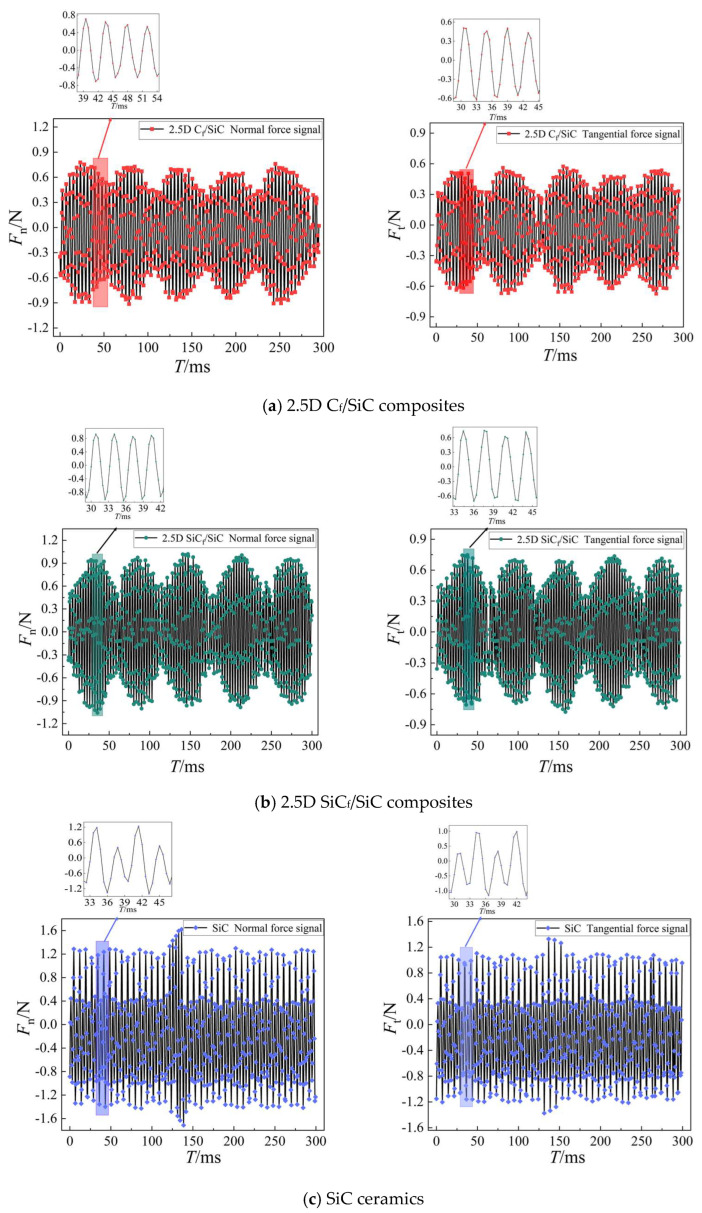
Real-time grinding force signals for the three materials under the same grinding parameters (*v*_s_ = 1.885 m/s, *a*_p_ = 9 μm, *v*_w_ = 120 μm/s).

**Figure 11:**
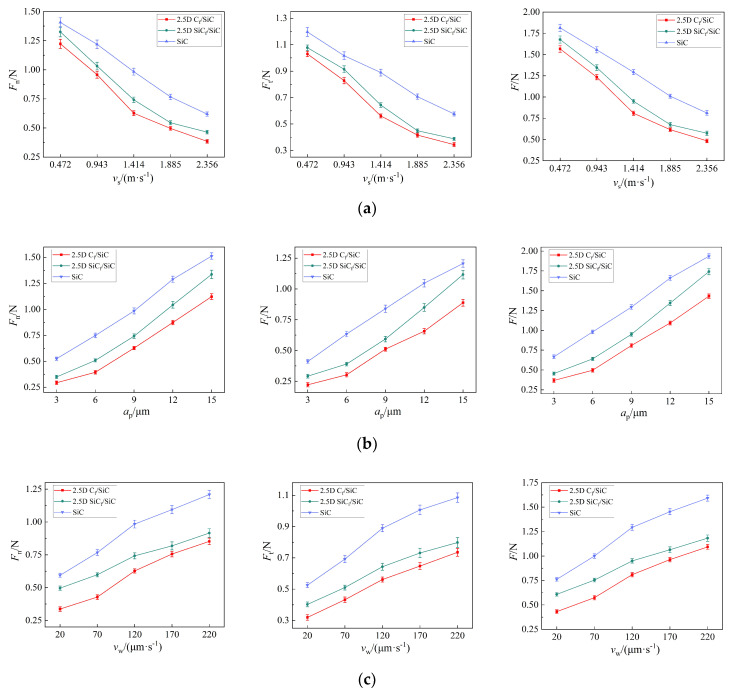
Influence of the grinding parameters (**a**) grinding speed *v*_s_; (**b**) grinding depth *a*_p_; and (**c**) feed speed *v*_w_ on the grinding force of the three materials.

During the micro-grinding process, the grinding forces of the three materials were measured using a Kistler 9257B three-dimensional dynamometer. The real-time signals of the measured normal grinding force *F*_n_ and tangential grinding force *F*_t_ are shown in Figure 10. Firstly, the average values of the two forces during the entire grinding process of a single experiment could be obtained through data processing. Then, similar to the previous treatment method for surface roughness, the average values of *F*_n_ and *F*_t_ were calculated for five parallel experiments. It was found that the deviations between the values of each experiment and their average values were within ±5%. The average values are as shown in Table 2. Finally, the average of the total grinding force *F* could be calculated from *F*_n_ and *F*_t_. For a clearer and more intuitive expression, *F*_n_, *F*_t_, and *F* are all plotted as line graphs in Figure 11.

For the three materials, the influences of grinding parameters on their micro-grinding forces are basically consistent. As shown in Figure 11a, the grinding force decreases with the increase in grinding speed v_s_. When vs. increases from 0.471 m/s to 2.356 m/s, the average total grinding force *F* of 2.5D C_f_/SiCs, 2.5D SiC_f_/SiCs, and SiC ceramics decreases by 69.1%, 65.8%, and 55.3%, respectively. Meanwhile, as shown in Figure 11b,c, the grinding force increases with the increase in grinding depth *a*_p_ and feed speed *v*_w_. When *a*_p_ increases from 3 μm to 15 μm, the *F* of the three materials increases by 288.9%, 282.6%, and 190.1%, respectively. When *v*_w_ increases from 20 μm/s to 220 μm/s, the *F* of the three materials increases by 153.6%, 94.6%, and 109.2%, respectively. Overall, it can be seen that among the three grinding parameters, *a*_p_ has the most significant impact on the grinding force. The variation trend of micro-grinding force with parameters are consistent with the results obtained from micro-grinding of dental bioceramics thread structures [29], ultrasonic vibration grinding 2.5D C_f_/SiC composites [30], and conventional grinding of SiC_f_/SiC composites [15]. However, in micro-grinding, the ratio of the normal force to the tangential force is around 1.3. In conventional grinding, this ratio is often greater than 2 or even close to 3 [15].

Under the same grinding parameters, the grinding force of 2.5D C_f_/SiCs is slightly smaller than that of 2.5D SiC_f_/SiCs. When the grinding parameters and the micro-grinding tools are the same, the material properties determine the magnitude of the grinding force since the grinding force is related to the material properties, grinding parameters, and geometric parameters of micro-grinding tools. For the two composite materials, their matrixes are both SiC ceramics and their content of reinforcing fibers are both around 37%. However, given that the hardness of SiC fibers is greater than that of C fibers, the average grinding force of 2.5D C_f_/SiCs is greater than that of 2.5D SiC_f_/SiCs under the same grinding parameters.

Additionally, as shown in Figure 11, under the same grinding parameters, the micro-grinding forces of the two composites are obviously lower than that of SiC ceramics. By comparing the real-time force signals in Figure 10, it can be observed that the real-time force signals of the two composites are relatively stable, while the signals of SiC ceramics exhibit a large number of spikes.

There are massive interfacial phases between the reinforced fibers and the matrix in 2.5D C_f_/SiCs and 2.5D SiC_f_/SiCs. The physical properties of the interface phase such as bonding strength and hardness are far lower than those of SiC ceramics. During the micro-grinding process, when the material is removed due to the action of the abrasive particles, a huge amount of energy is generated inside the material. The energies transmitted by crack propagation can be released and buffered when they reach the interfacial phases. The presence of the massive interfaces causes cracks to frequently deflect within the material, releasing a large amount of energy. The interfacial phases could be compared to the ‘flood discharge channels’ when the material fractures. Therefore, the micro-grinding forces of 2.5D C_f_/SiCs and 2.5D SiC_f_/SiCs are relatively stable and smaller than those of the SiC ceramics.

However, during the micro-grinding process of SiC ceramics, due to the absence of barriers from other phases in the material, some transverse and longitudinal cracks propagate deeper and farther into the material before their intersection. SiC ceramics are often removed in large chunks, leaving deep pits on the ground surface. During this process, a huge amount of energy will be accumulated, resulting in high grinding force and a large number of spikes in the real-time force signal. From the perspective of grinding force, the grindability of composites is better than that of SiC ceramics.

## 4. Conclusions

In this research, the micro-grinding processes and performance of 2.5D C_f_/SiCs, 2.5D SiC_f_/SiCs, and SiC ceramics were investigated. Differences in surface microstructure, surface roughness, and grinding force of the three materials were comparatively analyzed from the perspective of material structure, crack propagation, energy transmission, etc. Our main conclusions are summarized as follows:

(1) The presence of reinforcing fibers and interfacial phases in both composite materials inhibits crack propagation during micro-grinding. The interruption and frequent deflection of cracks make their surfaces after micro-grinding flat with small defects. However, because of the absence of barriers from other phases, the random propagation of cracks in SiC ceramics is severe. Some cracks extend further and deeper below the surface. After the material is removed, some serious pit defects are left on the ground surface.
(2) The micro-grinding process of 2.5D C_f_/SiCs is similar to that of 2.5D SiC_f_/SiCs, both of which are related to the fiber direction. There are differences in crack propagation paths and fiber fracture positions in different fiber directions. This makes the weft fiber layer prone to defects such as fiber pullout, fiber outcrop, and interfacial debonding, while the radial fiber layer is prone to defects such as fiber cracks, interfacial debonding, and fiber stripping.
(3) For these three materials, the influence of grinding parameters on surface roughness *Ra* is basically consistent. *Ra* decreases with the increase in grinding speed *v*_s_, while it increases with the increase in grinding depth *a*_p_ and feed speed *v*_w_. Under the same grinding parameters, the *Ra* of the three materials after micro-grinding is SiC ceramics > 2.5D SiC_f_/SiCs > 2.5D C_f_/SiCs.
(4) During the micro-grinding process, the energies generated inside the composite materials can be released and buffered when the crack expands to the interfacial phases. Therefore, the real-time grinding force signals of 2.5D C_f_/SiCs and 2.5D SiC_f_/SiCs are relatively stable. For SiC ceramics, a large amount of energy will be accumulated during the process of severe crack propagation and the generation of pit defects. Thus, there are sharp spikes in their real-time grinding force signals. Under the same grinding parameters, the average grinding force of the three materials is: SiC ceramics > 2.5D SiC_f_/SiCs > 2.5D C_f_/SiCs.

## Figures and Tables

**Table 1:** Grinding parameters in the single-factor experiment.

	Group No.	1	2	3
Factors				
Grinding speed *v*_s_ (m/s)		0.471, 0.942, 1.414, 1.885, 2.356	1.414	1.414
Grinding depth *a*_p_ (μm)		9	3, 6, 9, 12, 15	9
Feed speed *v*_w_ (μm/s)		120	120	20, 70, 120, 170, 220

**Table 2:** Single-factor experimental results for micro-grinding of the three materials.

Experiment No.	Grinding Parameters			2.5D C_f_/SiC Composites			2.5D SiC_f_/SiC Composites			SiC Ceramics		
	*v*_s_ (m/s)	*a*_p_ (μm)	*v*_w_ (μm/s)	*F*_n_/N	*F*_t_/N	*Ra*/μm	*F*_n_/N	*F*_t_/N	*Ra*/μm	*F*_n_/N	*F*_t_/N	*Ra*/μm
1	0.471	9	120	1.223	0.980	1.004	1.326	1.027	1.156	1.407	1.146	1.235
2	0.942	9	120	0.958	0.778	0.921	1.032	0.864	1.026	1.219	0.967	1.074
3	1.414	9	120	0.627	0.511	0.805	0.742	0.593	0.878	0.984	0.839	0.927
4	1.885	9	120	0.496	0.366	0.663	0.545	0.399	0.795	0.767	0.657	0.846
5	2.356	9	120	0.385	0.294	0.587	0.464	0.338	0.636	0.619	0.526	0.690
6	1.414	3	120	0.293	0.222	0.501	0.349	0.292	0.555	0.525	0.411	0.638
7	1.414	6	120	0.394	0.303	0.622	0.508	0.390	0.695	0.748	0.634	0.792
8	1.414	9	120	0.627	0.511	0.805	0.742	0.593	0.878	0.984	0.839	0.927
9	1.414	12	120	0.873	0.657	0.916	1.043	0.849	1.006	1.289	1.046	1.106
10	1.414	15	120	1.122	0.888	1.102	1.336	1.116	1.269	1.513	1.206	1.356
11	1.414	9	20	0.337	0.269	0.632	0.496	0.352	0.684	0.594	0.475	0.747
12	1.414	9	70	0.428	0.382	0.729	0.598	0.460	0.771	0.767	0.642	0.852
13	1.414	9	120	0.627	0.511	0.805	0.742	0.593	0.878	0.984	0.839	0.927
14	1.414	9	170	0.756	0.597	0.922	0.818	0.681	0.963	1.094	0.956	0.989
15	1.414	9	220	0.852	0.685	0.985	0.916	0.748	0.990	1.209	1.035	1.020

## Data Availability

The data presented in this study are available on request from the corresponding author.
